# Comparison of Methods to Measure Methane for Use in Genetic Evaluation of Dairy Cattle

**DOI:** 10.3390/ani9100837

**Published:** 2019-10-21

**Authors:** Philip C. Garnsworthy, Gareth F. Difford, Matthew J. Bell, Ali R. Bayat, Pekka Huhtanen, Björn Kuhla, Jan Lassen, Nico Peiren, Marcin Pszczola, Diana. Sorg, Marleen H.P.W. Visker, Tianhai Yan

**Affiliations:** 1 School of Biosciences, University of Nottingham, Sutton Bonington Campus, Loughborough LE12 5RD, UK; Matthew.Bell@nottingham.ac.uk; 2 Department of Molecular Biology and Genetics—Center for Quantitative Genetics and Genomics, Aarhus University, Blichers Allé 20, 8830 Tjele, Denmark; garethdifford@gmail.com (G.F.D.); jan.lassen@mbg.au.dk (J.L.); 3 Animal Breeding and Genomics, Wageningen University & Research, P.O. Box 338, 6700 AH Wageningen, The Netherlands; marleen.visker@frieslandcampina.com; 4 Milk Production, Production Systems, Natural Resources Institute Finland (Luke), FI 31600 Jokioinen, Finland; alireza.bayat@luke.fi; 5 Department of Agricultural Research for Northern Sweden, Swedish University of Agricultural Sciences, SE-901 83 Umeå, Sweden; Pekka.Huhtanen@slu.se; 6 Institute of Nutritional Physiology “Oskar Kellner”, Leibniz Institute for Farm Animal Biology (FBN), Wilhelm-Stahl-Allee 2, 18196 Dummerstorf, Germany; b.kuhla@fbn-dummerstorf.de; 7 Flanders Research Institute for Agriculture, Fisheries and Food, Animal Sciences Unit, Scheldeweg 68, 9090 Melle, Belgium; Nico.Peiren@ilvo.vlaanderen.be; 8 Department of Genetics and Animal Breeding, Poznan University of Life Sciences, Wolynska 33, 60-637 Poznan, Poland; mbee@up.poznan.pl; 9 Institute of Agricultural and Nutritional Sciences, Martin Luther University Halle-Wittenberg, Animal Breeding, Theodor-Lieser-Str. 11, 06120 Halle, Germany; dianasorg@yahoo.de; 10 German Environment Agency (Umweltbundesamt), Wörlitzer Platz 1, 06844 Dessau-Roßlau, Germany; 11 Agri-Food and Biosciences Institute, Hillsborough, Co. Down BT26 6DR, UK; Tianhai.Yan@afbini.gov.uk

**Keywords:** methane, dairy cows, genetic evaluation, greenhouse gases, environment

## Abstract

**Simple Summary:**

Methane is a greenhouse gas with a global warming potential 28 times that of CO_2_. Enteric methane accounts for 17% of global methane emissions and 3.3% of total global greenhouse gas emissions from human activities. There is, therefore, significant research interest in finding ways to reduce enteric methane emissions by ruminants. Partners in Expert Working Group 2 (WG2) of the European Cooperation in Science and Technology (COST) Action METHAGENE have used several methods for measuring methane output by individual dairy cattle under various environmental conditions. Methods included respiration chambers, the sulphur hexafluoride (SF_6_) tracer technique, breath sampling during milking or feeding, the GreenFeed system, and the laser methane detector. Respiration chambers are considered the ‘gold standard’, but are unsuitable for large-scale measurements of methane emissions, which are needed for genetic evaluations. In this study, the suitability of methods for large-scale studies was reviewed and compared. All methods showed high correlations with respiration chambers, but comparisons among alternative methods generally had lower correlations. Results confirm, however, that there is sufficient correlation between methods for measurements from all methods to be combined, with appropriate weightings, for use in international genetic studies. This will pave the way for breeding cattle with lower methane emissions.

**Abstract:**

Partners in Expert Working Group WG2 of the COST Action METHAGENE have used several methods for measuring methane output by individual dairy cattle under various environmental conditions. Methods included respiration chambers, the sulphur hexafluoride (SF_6_) tracer technique, breath sampling during milking or feeding, the GreenFeed system, and the laser methane detector. The aim of the current study was to review and compare the suitability of methods for large-scale measurements of methane output by individual animals, which may be combined with other databases for genetic evaluations. Accuracy, precision and correlation between methods were assessed. Accuracy and precision are important, but data from different sources can be weighted or adjusted when combined if they are suitably correlated with the ‘true’ value. All methods showed high correlations with respiration chambers. Comparisons among alternative methods generally had lower correlations than comparisons with respiration chambers, despite higher numbers of animals and in most cases simultaneous repeated measures per cow per method. Lower correlations could be due to increased variability and imprecision of alternative methods, or maybe different aspects of methane emission are captured using different methods. Results confirm that there is sufficient correlation between methods for measurements from all methods to be combined for international genetic studies and provide a much-needed framework for comparing genetic correlations between methods should these become available.

## 1. Introduction

Methane is a greenhouse gas with a global warming potential 28 times that of CO_2_ [1]. Methane from ruminant livestock is generated during microbial fermentation in the rumen and hindgut (enteric methane), and from decomposition of manure. Enteric methane contributes 80% of methane emissions by ruminants, and manure decomposition contributes 20%. Enteric methane accounts for 17% of global methane emissions and 3.3% of total global greenhouse gas emissions from human activities [2]. There is, therefore, a significant research interest in finding ways to reduce enteric methane emissions by ruminants.

Ruminant animals have evolved with a digestive system to digest plant materials efficiently. Like most mammals, ruminants lack the cellulase enzyme required to break the beta-glucose linkages in cellulose, but they play host to diverse populations of rumen microbes that can digest cellulose and other plant constituents. When rumen bacteria, protozoa and fungi ferment carbohydrates and proteins in plant materials, they produce volatile fatty acids, principally acetate, propionate and butyrate. High-fibre diets favour acetate synthesis. Synthesis of acetate and butyrate are accompanied by release of metabolic hydrogen, which, if allowed to accumulate in rumen fluid, has negative effects on microbial growth, and feed digestibility [3]. Rumen Archaea are microorganisms that combine metabolic hydrogen with CO_2_ to produce methane and water. Archaea play a vital role, therefore, in protecting the rumen from excess metabolic hydrogen, and the methane they produce is an inevitable product of rumen fermentation.

The amount of methane produced by a ruminant animal is related to the amount of organic matter digested in the rumen, particularly the fibre fraction, and hence the amount of acetate and metabolic hydrogen produced. The main determinants of daily methane production, therefore, are dry matter intake and diet composition: the more feed consumed, and/or the greater the fibre content of the diet, the more methane is produced per day. Nutritional approaches for methane mitigation include reducing the forage to concentrate ratio of diets, increasing dietary oil content, and dietary inclusion of rumen modifiers and methane inhibitors [2,4,5]. Some of these mitigation strategies act through reductions in forage digestibility or feed intake, which can have negative consequences for feed efficiency and methane output per kg of product. Methane output per kg of product is affected mainly by milk yield or growth rate per animal, and by herd-level factors, such as fertility, disease incidence and replacement rate, which affect not only the milk yield of the herd, but also methane produced by replacement animals [6]. Even when all these influences have been taken into account, methane output varies considerably between individual animals. During development of the Metabolisable Energy system, thousands of determinations of methane production by sheep and cattle were performed at the Rowett Research Institute, Aberdeen, UK, using animals fed a restricted amount of feed in closed-circuit respiration chambers. Under these carefully controlled conditions, for animals fed on the same feed, the between-animals coefficient of variation (CV) in methane was 8.1% [7]. Analysis of 1335 records of methane production by cattle fed on a variety of diets in respiration chambers showed a strong relationship (r^2^ = 0.91) between methane production and dry matter intake [8]. At the average dry matter intake, however, there was a twofold difference in methane production between the lowest and highest emitters, and at the average methane production rate there was a twofold difference in dry mater intake. These two studies illustrate the range in variation among individual animals that may be encountered under research conditions.

Researchers view individual variation in different ways, according to the aims of their research. When comparing treatments, or evaluating the nutritional value of diets, investigators want to minimise between-animal variation so as to maximise the power of their study, minimise the number of animals required, and increase the chance of detecting a significant difference between treatments. When evaluating populations for genetic studies, on the other hand, variation between animals is of interest, so the aim is to quantify variation, which can then be partitioned into genetic and environmental components. These components are used to determine the amount of variation that is heritable, and genetic correlations between traits currently used in breeding and a possible new trait like methane production. This enables the breeder to decide if there is any merit to adding the new trait to the breeding goal. In terms of methane mitigation, nutritional and management approaches provide greater short-term reductions, but genetic approaches provide greater long-term reductions because genetic improvements are cumulative and permanent. All disciplines require a measurement to be suitably accurate and precise to conduct hypothesis testing and draw reasonable inferences with a given level of certainty. However, several factors influence the choice of the most suitable measurement method such as cost, level of accuracy, precision, scope of application, and scale, which vary across disciplines [9]. For instance, genetic selection programs require methane measurements on thousands of related individuals under the environmental conditions in which the animals are expected to perform [10]. This can be challenging because dairy cattle perform in a wide range of conditions (e.g., grazing vs indoor housing).

Respiration chambers are calibrated to be accurate and precise, and are the gold standard for benchmarking new methods. Where an alternative method may be cheaper, less invasive, easier to implement, or have a wider scope of applications, it is of value to assess the relative accuracy, precision and correlation with the gold standard to assess the relative worth of the alternative method [11]. All methods measure methane with some level of error, so the ‘true value’ of an individual is not known. However, when the level of measurement error increases, so too does the imprecision. When comparing two methods where one or both methods has high imprecision a phenomenon known as ‘attenuation of errors’ occurs [12]. The increased measurement error biases the correlation between the two methods downwards and reduces the efficacy of detecting significant differences in accuracy [13]. Or in terms of linear regression terms, when the observed CV of an alternative method is higher than that of the gold standard method, the slope of regression between the methods is decreased and the intercept is biased upwards [14,15].

A variety of technologies are being developed and employed to measure methane emissions by individual dairy cattle under various environmental conditions, as is evidenced by frequent reviews (e.g., [9,16,17]). The aim of the current study was to review and compare the suitability of methods for large-scale measurements of methane output by individual animals, which may be combined with other databases for genetic evaluations. Comparisons included assessing the accuracy, precision and correlation between methods. Combining datasets from different countries and research centres could be a successful strategy for making genetic progress in a trait that is difficult to measure, such as methane emissions if the methods are correlated [17]. Potential for combining large-scale data is of particular interest because data sharing could lead to powerful international collaborations and efficient sharing of resources. Accuracy and precision of methods are important, but data from different sources can be appropriately weighted or adjusted when combined, so any methods can be combined if they are suitably correlated with the ‘true’ value. The objective of the current study, therefore, was to examine correlations among results obtained by different methods, ultimately leading to an estimate of confidence limits for selecting individual animals that are high or low emitters.

## 2. Methods for Measuring Methane

Partners in Expert Working Group 2 (WG2) of the European Cooperation in Science and Technology (COST) Action METHAGENE (www.methagene.eu) have used a variety of methods for measuring methane output by individual animals. Methods include respiration chambers, the sulphur hexafluoride (SF_6_) tracer technique, breath sampling during milking or feeding, the GreenFeed system, and the laser methane detector. Each method measures different components of methane output. Only respiration chambers measure total emissions from the animal via the oral, nasal and anal routes; all other methods ignore emissions via the anus and only measure methane emitted in breath. Breath measurements are justified because 99% of methane is emitted from the mouth and nostrils, and only 1% via the anus [18]. The SF_6_ technique samples breath over 24 h, whereas other techniques use spot samples of breath over periods of minutes throughout the day, so diurnal variation has to be considered. The majority of methane (87%) is released by eructation [18,19], which provides a clear signal for sample processing. 

The main features of methods for measuring methane output by individual animals are summarised in Table 1. Values for each feature are based on the experience of experts in METHAGENE WG2 who have used the methods. All values are relative, and somewhat subjective, because absolute values will depend on installation and implementation of each method at different research centres. Each method is described and discussed in more detail in the next five sub-sections.

### 2.1. Respiration Chamber

Respiration chambers for open- or closed-circuit indirect calorimetry are considered the ‘Gold Standard’, and were used extensively in nutrition studies when establishing the Metabolisable Energy system [7]. A single animal (or occasionally more) is confined in a chamber for between 2 and 7 days. Concentration of methane (and other gases if required) is measured at the air inlet and outlet vents of the chamber. The difference between outlet and inlet concentrations is multiplied by airflow to indicate methane emissions rate. In most installations, a single gas analyser is used to measure both inlet and outlet concentrations, often for two or more chambers. This involves switching the analyser between sampling points at set intervals, so concentrations are actually measured for only a fraction of the day. 

Respiration chambers vary in construction materials, size of chamber, gas analysis equipment and airflow rate, all of which can influence results. Validation of 22 chambers at six UK research sites revealed an uncertainty of 25.7% between facilities, which was reduced to 2.1% when correction factors were applied to trace each facility to the international standard for methane [20]. The main sources of uncertainty were stability and measurement of airflow, which are crucial for measuring methane emission rate. The authors concluded, however, that chambers were accurate for comparing animals measured at the same site. It is an added challenge, when benchmarking alternative methods against respiration chambers, that the respiration chambers themselves have not been benchmarked against respiration chambers at other facilities. 

For large-scale evaluation of methane emissions by individual animals, respiration chambers are challenging, with only a single study in growing Angus steers and heifers exceeding 1000 animals, which found methane production to be moderately heritable h^2^ = 0.27 ± 0.07 [21]. Installation costs and running costs are high, and only one animal can be measured at a time. If the monitoring time is three days per animal, and chambers are run continuously, then maximum throughput would be approximately 100 animals per chamber per year. In practice, throughput is likely to be 30 to 50 animals per year. Cows are social animals, and confinement in a chamber may ultimately influence their feeding behaviour, resulting in less feed being consumed and in a different meal pattern compared with farm conditions. Altered feeding patterns or levels is not a problem for metabolic studies evaluating feeds, but can be a problem when evaluating individual animals. Furthermore, the representativeness of respiration chambers to grazing systems has been called into question [22]. However, promising developments have led to more animal friendly respiration chambers constructed from cheaper, transparent materials. These lower the cost and reduce the stress of confinement with minimal disruptions to accuracy, precision and no drop in feed intake of the cows [23]. 

### 2.2. The SF_6_ Technique

The SF_6_ tracer gas technique was developed in an attempt to measure methane emissions by animals without confinement in respiration chambers [24]. Air is sampled near the animal’s nostrils through a tube attached to a halter and connected to an evacuated canister worn around the animal’s neck or on its back. A capillary tube or orifice plate is used to restrict airflow through the tube so that the canister is between 50 and 70% full after approximately 24 h. A permeation tube containing SF_6_ is placed into the rumen of each animal. The pre-determined release rate of SF_6_ is multiplied by the ratio of methane to SF_6_ concentrations in the canister to calculate methane emission rate.

Many research centres have used the SF_6_ technique with variations in design of sampling and collection equipment, permeation tubes, and gas analysis [25]. Reliable results depend on following standard protocols, with greatest variation coming from accuracy of determining SF_6_ release rate from permeation tubes and control of sampling rate. With capillary tubes, sampling rate decreases as pressure in the canister increases, whereas an orifice plate gives a steadier sampling rate over 24 h [26]. A source of error that has not been evaluated is that animals might interact and share methane emissions when the sampling tube of one animal is near the head of another animal. There is good agreement between methane emissions measured by the SF_6_ technique and respiration chambers, although results from the SF_6_ technique are more variable [27,28]. 

For large-scale evaluation of methane emissions by individual animals, the SF_6_ technique is more useful than respiration chambers. Animal behaviour and intake might be affected by wearing the apparatus, and by daily handling to exchange canisters, but the technique is considerably less intrusive than respiration chambers, because cows remain in the herd. Labour and monetary costs for changing canisters each day and for lab analysis are high. Throughput is limited by the number of sets of apparatus available, handling facilities, labour, and the capacity of the lab for gas analysis. Animals need to be measured for 5 to 7 days, and it is recommended that group size should be less than 15 animals [25], so maximum throughput would be about 750 animals per year. Heritability has been estimated for methane production in grazing Holstein cows at h^2^ = 0.33 ± 0.15 [29].

### 2.3. Breath Sampling during Milking and Feeding

Several research groups have developed methods to measure methane concentration in breath of cows during milking and/or feeding. These are often referred to as ‘sniffer methods’ because they use devices originally designed to detect dangerous gas leaks. Air is sampled near the animal’s nostrils through a tube fixed in a feed bin and connected directly to a gas analyser. The feed bin might be in an automatic milking station [14,30,31,32,33] or in a concentrate feeding station [34]. Different research centres use different gas analysers (Nondispersive Infrared (NDIR), Fourier-transform infrared (FTIR) or photoacoustic infrared (PAIR)) and different sampling intervals (1, 5, 20 or 90–120 s). Methane concentration during a sampling visit of typically between 3 and 10 min may be specified as the overall mean, or the mean of eructation peaks. Some centres use CO_2_ as a tracer gas and calculate daily methane output according to ratio of methane to CO_2_ and daily CO_2_ output predicted from performance of the cow [35]. Repeatability and rank correlations were higher for eructation peaks than for mean concentrations, and were higher for eructation peaks than for methane to CO_2_ ratio [36]. However, all methods show good repeatability.

For large-scale evaluation of methane emissions by individual animals, breath-sampling methods have significant advantages compared with other methods. Breath-sampling methods are non-invasive because, once installed, animals are unaware of the equipment and are in their normal environment. Animals follow their normal routine, which includes milking and feeding, so no training of animals, handling, or change of diet is required. Equipment is relatively cheap, although more expensive gas analysers are available, and running costs are negligible. 

The compromise for non-invasiveness of breath-sampling is that concentrations of gasses in the sampled air are influenced by cow head position relative to the sampling tube [15]. The use of head position sensors and data filtering algorithms can remove the effects when the cow’s head is completely out of the feed bin [37], but not within the feed bin. Consequently, sniffer measurements are more variable than flux methods, with factors like variable air flow in the barn increasing measurement error (imprecision), and head position, a highly repeatable characteristic, inflating between-cow variability. 

Using CO_2_ as a tracer gas partly addresses the issue but, because CO_2_ arises from metabolism as well as rumen fermentation, variability of CO_2_ emissions has to be considered. A further consideration is diurnal variation in breath concentrations of methane and CO_2_ because animals are spot-sampled at different times of day and night. Diurnal variation can be accounted for either by fitting a model derived from the whole group of animals, or by including time of measurement in the statistical model [30]. 

The number of observations per analyser is limited only by number of cows assigned to one automatic milking station or concentrate feeding station and length of time equipment is installed. Typically, each analyser will record 40 to 70 animals 2 to 7 times per day for 7 to 10 days, although the number of sampling stations per analyser can be increased by using an automatic switching system [32]. Throughput per analyser is likely to be 2000 to 3000 animals per year. Estimates of heritability for methane production measured using this method range from h^2^ = 0.12 to 0.45 over multiple studies [38,39].

### 2.4. GreenFeed

GreenFeed (C-Lock Inc., Rapid City, SD, USA) is a sophisticated sniffer system where breath samples are provided when animals visit a bait station [15]. As with other sniffer systems, GreenFeed samples breath from individual animals several times per day for short periods (3 to 7 min). GreenFeed is a portable standalone system used in barn and pasture applications, and incorporates an extractor fan to ensure active airflow and head position sensing for representative breath sampling [9]. Measurements are pre-processed by the manufacturer, and data are available in real time through a web-based data management system [40]. As GreenFeed captures a high proportion of emitted air and measures airflow, which can be calibrated using a tracer gas, methane emission is estimated as a flux at each visit. Providing visits occur throughout the 24 h, methane emission can be estimated directly as g/day [15,40]).

A limitation of the GreenFeed system is that animals require training to use the system, although animals which have been trained to use the system will readily use it again [41]. However, some animals will not use the system or will use it infrequently, and frequency of visits is affected by diet [42]. This can be a challenge when screening commercial herds for methane emission under genetic evaluation. 

The manufacturer recommends 15 to 25 animals per GreenFeed unit, and recordings are made typically for 7 days. If all animals visit the unit adequately, throughput per unit is likely to be 750 to 1250 animals per year.

### 2.5. Laser Methane Detector

The laser methane detector (LMD) is a highly responsive, hand-held device that is pointed at an animal’s nostrils and measures methane column density along the length of the laser beam (ppm.m). In the first implementation of LMD on a farm, measurements for each cow were taken over periods of 15 to 25 s between eructation events, and could detect methane emitted each time the animal breathed out [43]. In a later study with sheep and beef cattle, monitoring periods of 2 to 4 min allowed authors to separate breathing cycles from eructation events [44]. Typically, animals are restrained either manually or in head yokes at a feed fence for the required length of time. The operator has to stand at the same distance (1 to 3 m) from each animal every time and must be careful to keep the laser pointed at the animal’s nostrils throughout the measurement period. 

The LMD can be used in the animal’s normal environment, although for consistency restraint is required during measurement. Because the LMD measures methane in the plume originating from the animal’s nostrils, results can be affected by factors such as: distance from the animal; pointing angle; animal’s head orientation and head movement; air movement and temperature in the barn; adjacent animals; and operator variation [45]. Operator variation is likely to be one of the biggest factors, because the operator controls distance and pointing angle, and is responsible for ensuring that the laser remains on target. The structure of the barn and the resulting ventilation conditions and wind speed at the location of the measurement are also considerable sources of variation in recorded methane.

Assuming operator fatigue does not limit measurements, each LMD could record up to 10 animals per hour. If each animal is recorded 3 times (on 3 consecutive days, for example, as in [46]), throughput is likely to be up to 1000 animals per year.

## 3. Agreement between Methods

In method comparison studies, simultaneous repeated measures per cow with two or more methods are required in order to assess systematic differences between methods (means) and random differences (precision) and correlation between methods free of residual error. Furthermore, short time differences between repeated measures per subject are needed to ensure that the underlying biology of the cow has not changed. Not all methods can be recorded simultaneously, and the methane emission of cows changes both throughout the day and over the lactation period. In such instances, either cross-over designs are needed, or else matched-pair repeated measures designs. Members of METHAGENE WG2 provided data from studies in which two or more methods had been used to measure methane output (g/day) by individual dairy cows. Methods had been applied to each cow either concurrently or consecutively within a short timeframe.

Seven main methods were represented: respiration chambers; SF_6_; GreenFeed; LMD; and three breath-sampling systems based on different gas analysers. Gas analysers incorporated different technologies to measure methane: NDIR (e.g., Guardian Plus, Edinburgh Instruments, Edinburgh, UK), FTIR (e.g., Gasmet 4030, Gasmet Technologies Oy, Helsinki, Finland), and PAIR (e.g., F10, Gasera Ltd., Turku, Finland). In the contributing studies, NDIR and FTIR were used in automatic milking stations, and PAIR was used in concentrate feeding stations. One NDIR study and all FTIR and PAIR studies used CO_2_ as a tracer gas, with daily CO_2_ output calculated either from milk yield, live weight and days pregnant (t1) or from metabolisable energy intake (t2). Two NDIR studies were based on methane concentration in eructation peaks rather than mean methane concentration, so were treated as separate methods. By separating NDIR studies, a total of 8 distinct methods were available, giving a matrix of 28 potential combinations for comparisons. Data were available for 13 method combinations.

Method comparisons were conducted using bivariate models (repeatability animal models) to obtain correlations between ‘true values’, also known as repeated measures correlations or individual level correlations [33,47]. Variance components, including between-cow variation and within-cow variation (precision) and means (accuracy), were used in the calculation of between-cow coefficient of variation (CV, %) and total CV and repeatability (Table 2). Where single measurements were available for each method Pearson’s correlation was reported and where repeated measures per subject were available repeated measures correlation was reported. Lin’s concordance correlation coefficient [48] was calculated for each method comparison.

Respiration chambers were the most precise method, as can be seen by the smaller between-cow CV% and total CV compared to alternative methods, and respiration chambers are by definition the most accurate (Table 2). All methods tested showed high correlations with respiration chambers but none of the correlations exceeded 0.90. This is in part due to the increased imprecision of alternative methods, as even the most accurate and precise method will compare poorly to a less precise method. These correlations are also likely to be underestimated because none of the methods could be recorded simultaneously with respiration chambers and had to be recorded in cross-over designs. Consequently, the true value for each cow may have changed due to changes in the underlying biology of the cow over time between measurements. 

For the methods with repeated measures per cow, the two mass flux methods, SF_6_ and GreenFeed, had the highest repeated measures correlations (0.87 ± 0.08 and 0.81 ± 0.10), which outperformed the concentration-based NDIR method using CO_2_ tracer gas method t1. Of the two concentration methods evaluated against respiration chambers using single measurements, NDIR Peaks had a higher correlation (0.89 ± 0.07) than the PAIR CO_2_ tracer gas method t2 (0.80 ± 0.10). 

Comparisons among alternative methods generally had lower correlations than comparisons with respiration chambers, despite having relatively higher numbers of animals, and in most cases simultaneous or near simultaneous repeated measures per cow per method. This could be due to the increased variability and imprecision of alternative methods, as seen by the increased CVs or due to the possibility that different aspects of methane emission are captured using different methods. The study of [49] comparing SF_6_ and GreenFeed reported a low Pearson correlation of 0.40, despite having a large number of animals with repeated measures per method, the authors appear not to have estimated a repeated measures correlation, which could be larger. Estimating a repeated measures correlation between these two mass flux methods is a priority as it would clarify the inexplicable disagreement between two methods which both correlate highly with the gold standard method. With the exception of the aforementioned study, the imprecision was low in the mass flux measure comparisons as compared to the concentration-based methods [50]. Two of the sniffer methods evaluated, FTIR CO_2_t1 and NDIR CO_2_t1, correlated close to unity (0.97), most likely due to the shared prediction equation for CO_2_ tracer gas. Nevertheless, all correlations derived from actual data were positive. This suggests that combination of datasets obtained with different methods is a realistic proposition for genetic studies. Calculation of adjustment or weighting factors for bias, accuracy and precision is beyond the scope of the current study, but would improve the value of combined datasets.

## 4. Conclusions

Measuring methane on large numbers of cows is a challenge. The high costs and low throughput of respiration chambers restrict their use to research studies measuring methane emissions on small numbers of individual animals. Respiration chambers remain the gold standard method, but benchmarking alternative methods against respiration chambers is challenging, because simultaneous replicate measures per cow are not feasible. Methods like SF_6_ and GreenFeed require lower capital investment and running costs than Respiration Chambers, and have higher throughput and potential for use in extensive and grazing situations, but costs are still prohibitive for recording large numbers of animals. Methods based on concentration are less precise and accurate than flux methods, but they are viable for large-scale measurement, which is a prerequisite of genetic evaluations. Further development is needed to increase the accuracy and precision of concentration methods. Several reviews of methods for measuring methane have made qualitative judgments based on individual comparison studies without expanding scope to genetic evaluations and considering repeated measure correlations between methods as proxies for genetic correlations. Results confirm that there is sufficient correlation between methods for measurements from all methods to be combined for international genetic studies and provide a much-needed framework for comparing genetic correlations between methods should these be made available.

## Figures and Tables

**Table 1:** Summary of the main features of methods for measuring methane output by individual animals ^1^.

Method	Purchase Cost ^2^	Running Costs ^2^	Labour ^2^	Repeatability	Behaviour Alteration ^3^	Throughput
Respiration chamber	High	High	High	High	High	Low
SF_6_ technique	Medium	High	High	Medium	Medium	Medium
Breath sampling during milking and feeding	Low ^4^	Low	Low	Medium	None	High
GreenFeed	Medium	Medium	Low	Medium	Low	Medium
Laser methane detector	Low	Low	High	Low	Low-Medium	Medium

^1^ Consensus views based on experiences of METHAGENE WG2 members. ^2^ Per measuring unit or group of animals. ^3^ Compared to no methane recording: low = measuring in situ; medium = some handling, training or change in routine; high = confinement. ^4^ Medium if using FTIR analyser.

**Table 2:** Comparisons of methods for recording methane emission in dairy cattle.

Alternate Methods ^1^ Versus Respiration Chambers								
Method	N Cows	N Obs	Mean S.E.	Rep S.E.	Between-Cow CV ^2^	Total CV	Correlation ^3^ (S.E.)	CCC ^4^ (S.E.)
SF_6_	33	97	471 (14.3)	0.44 (0.13)	11.6	17.4	0.87 (0.08)	0.30 (0.17)
Respiration Chambers	33	97	437 (10.7)	0.36 (0.08)	8.4	14.0		
GreenFeed	27	63	433 (8.7)	0.64 (0.08)	12.8	15.9	0.81 (0.10)	0.41 (0.12)
Respiration Chambers	27	63	459 (6.5)	0.51 (0.09)	8.1	11.3		
NDIR Peaks	12	12	376 (12.1)	N/A	N/A	11.1	0.89 (0.07)	0.88 (0.10)
Respiration Chambers	12	12	377 (10.7)	N/A	N/A	9.4		
NDIR CO_2_ t1	20	60	573 (16.8)	0.58 (0.11)	10.1	13.1	0.72 (0.11)	0.38 (0.21)
Respiration Chambers	20	60	521 (13.7)	0.61 (0.12)	9.1	11.7		
PAIR CO_2_ t2	21	21	555 (21.3)	N/A	N/A	11.3	0.80 (0.08)	0.70 (N/A)
Respiration Chambers	21	21	585 (14.1)	N/A	N/A	17.1		
Alternate methods ^1^ versus Alternate methods								
SF_6_	48	144	405 (22.5)	N/A	N/A	38.5	0.40 (0.18)	0.34 (N/A)
GreenFeed	48	144	373 (13.9)	N/A	N/A	25.8		
LMD	11	88	432 (24.8)	0.21 (0.11)	19.4	42.7	0.77 (0.23)	0.18 (0.23)
GreenFeed	11	88	423 (18.5)	0.49 (0.12)	11.4	16.8		
NDIR CO_2_ t1	27	63	586 (19.4)	0.59 (0.13)	13.2	17.2	0.64 (0.18)	0.14 (0.19)
GreenFeed	27	63	453 (9.8)	0.75 (0.08)	9.7	11.2		
NDIR CO_2_ t1	39	118	365 (8.3)	0.66 (0.11)	13.9	17.1	0.60 (0.11)	0.18 (0.19)
LMD	39	118	363 (10.3)	0.14 (0.09)	7.5	19.6		
FTIR CO_2_ t2	34	68	315 (12.3)	0.77 (0.13)	21.3	24.3	0.57 (0.25)	0.20 (0.22)
LMD	34	68	299 (6.1)	0.27 (0.15)	7.5	14.5		
NDIR CO_2_ t1	45	90	383 (8.7)	0.85 (0.04)	14.0	15.2	0.58 (0.15)	0.14 (0.19)
NDIR Peaks	45	90	393 (8.1)	0.59 (0.09)	10.7	13.9		
FTIR CO_2_ t1	43	103	392 (8.1)	0.81 (0.05)	14.1	15.3	0.97 (0.02)	0.79 (0.12)
NDIR CO_2_ t1	43	103	382 (8.9)	0.86 (0.04)	12.2	13.6		
FTIR CO_2_ t1	45	90	392 (7.9)	0.81 (0.05)	12.2	13.6	0.53 (0.17)	0.15 (0.19)
NDIR Peaks	45	90	382 (8.2)	0.60 (0.09)	10.8	14.0		

^1^ SF_6_ = Sulphur hexafluoride tracer gas technique, LMD = Laser methane detector; NDIR = Nondispersive Infrared; FTIR = Fourier Transform Infrared; PAIR = Photoacoustic Infrared. CO_2_ t1 method uses CO_2_ predicted from milk yield, live weight and days pregnant; CO_2_ t2 method uses CO_2_ predicted from metabolisable energy intake. ^2^ Coefficient of variation (%). ^3^ When repeated measures per cow were made the repeated measures correlation was reported, when single measures per cow were made Pearson’s correlation was reported, N/A not available, due to single measurements. ^4^ Lin’s concordance correlation coefficient [48].
